# Viewing angle, skill level and task representativeness affect response times in basketball defence

**DOI:** 10.1038/s41598-024-53706-9

**Published:** 2024-02-09

**Authors:** Christian Vater

**Affiliations:** Institute of Sport Science, University of Bern, Bremgartenstrasse 145, 3012 Bern, Switzerland

**Keywords:** Psychology, Human behaviour

## Abstract

In basketball defence, it is impossible to keep track of all players without peripheral vision. This is the first study to investigate peripheral vision usage in an experimentally controlled setup, with sport-specific basketball stimuli from a first-person perspective, large viewing eccentricities (up to 90° to the left and right), and natural action responses. A CAVE and a motion-tracking system was used to project the scenarios and capture movement responses of high- and low-skilled basketball players, respectively. Four video conditions were created: (1) a simple reaction time task without crowding (only attackers), (2) a simple reaction time task with crowding (with attackers and defenders), (3) a choice-reaction time task where the player cutting to the basket eventually passed the ball to another player and (4) a game simulation. The results indicated eccentricity effects in all tests, a crowding effect in condition 2, and expertise differences in conditions 3 and 4 only. These findings suggest that viewing eccentricity has an impact on response times, that crowding is a limiting factor for peripheral perception in sports games, and that high-skilled but not low-skilled players can compensate for eccentricity effects in real game situations, indicating their superior positioning and perceptual strategies.

## Introduction

Basketball players need to perceive much information at the same time (e.g., ball, teammates, opponents, own position on the field). It seems, that this is only possible by using their peripheral vision, i.e., by processing information without looking at them (see^1^ for a review on peripheral vision), coupled with an optimal positioning and head orientation^2^. In many reviews or meta-analyses, it has been shown that high-skilled players or professionals use their foveal vision different than their lower skilled counterparts^3–6^. They typically have longer fixation durations and/or different fixation patterns. In contrast, little is known about expertise differences for the use of peripheral vision.

When looking at the empirical studies on peripheral vision and the applied methods, many studies used eye-tracking and interpreted not looking at specific information as an indicator for the use of peripheral vision^7–10^. The majority of findings from team sport studies are based on subjective verbal reports^11,12^ or studies that used simplified responses^13–16^ or unnatural (3rd-person or birds-eye) viewing perspectives^17,18^. Especially simplified responses seem to be critical because they lead to different perceptual strategies than natural action responses^19^. Thus, there is a need for experimentally controlled studies, with objective measures, and representative test settings. Therefore, in this study, real basketball situations were recorded with 360° cameras which (a) were recorded from a first-person perspective (centre player position), (b) included experimental manipulations of player positions to test viewing eccentricity effects and (c) required natural action responses from the players.

Peripheral vision is regarded as an essential element for visual perception, in sports^2,20^ as well as in everyday life^21^ and has many useful functionalities. It is far more than a “warning system” to detect dangerous threats^22^. It has been discussed that peripheral information processing guides our eye movements, has its advantages in time-pressure situations, and helps to orient oneself in space^2,20^. The latter is connected to the use of optic flow information, i.e., how fast objects are moving (increasing or decreasing in size) on the retina^23,24^. Moreover, peripheral vision allows one to monitor multiple pieces of information at the same time, sometimes more efficiently than using eye movements^25^. Critical for decision-making in sports, peripheral vision is sensitive to motion information and responsible for processing this information within 99% of the visual field^26^. As an example, detecting the movement onset of another player should be possible with peripheral vision^11^. Such situations are often found in team sports, with many players that need to be monitored. For example, in basketball, a centre player must keep all relevant players in his or her field of view, to quickly react to a player cutting to the basket.

In basketball as well as in any other team sport, it can be expected that the eccentricity (i.e., the viewing angle) of relevant players matters. This hypothesis mainly arises when looking at findings from vision science and optometry research. This research shows that the visual eccentricity of a target affects reaction times^1^. As an example^27^, found that response times increase by 1.8 ms per visual degree. That means, when reacting to stimuli located in peripheral vision at 90° eccentricity, response times should be 162 ms slower than in foveal vision^28^ tested this with two soccer player skill groups using the peripheral perception test of the Vienna Test System (Schuhfried GmbH, Moedling, Austria), where a button must be released when (peripheral) LED lights turn off. They compared peripheral reaction times at 0°, 90° and 180° eccentricity for expert and intermediate soccer players and found that response times increase with eccentricity (216 ms at 0° to 278 ms at 180°). Response times did not differ for the two expertise groups. If these eccentricity and expertise findings can also be found in sport-specific tasks will be tested in this study.

Another important finding from vision science that has been overlooked in sport science is the crowding effect^29^. It describes that visual perception of a peripheral target is impaired if the target is masked by so-called “flankers”^30,31^. Because of the visual complexity of team sport situations^32,33^—imagine a corner kick in football where most players are near the penalty area and the goalkeeper needs to identify the players of the opposing team (for an illustration see Fig. 2 in^34^)—it can be expected that crowding affects perceptual and maybe also motor performance. This too has never been tested in a sports setting with a representative task design but will be examined in the current study.

Taken together, there is so far no research that tested the ability to monitor multiple players with a representative experimental paradigm, a first-person viewing perspective, players at realistic visual eccentricities, and participants being able to realize natural action responses. This research gap will now be filled. In the first stage, realistic basketball scenarios will be captured with 360° video recordings from a first-person perspective (centre position). Experienced basketball players will stage the basketball plays for four conditions: (1) a simple reaction time task with either of four offensive players cutting to the basket from different eccentricities; (2) the same situations as in condition 1 but with four defenders (to simulate natural crowding conditions), (3) the same situations as in test 2 but as a choice reaction time task where the player in possession of the ball eventually plays an extra pass and (4) natural gameplay with varying number of passes. Thus, the last condition will include natural motion cues and playing patterns, which skilled players have been shown to use more effectively than low-skilled players^3,35^.

Based on the empirical evidence for response time effects and the absence of eccentricity effects on response accuracy as a function of eccentricity, the following research questions and hypotheses only address response time as the main dependent variable. Nevertheless, potential speed-accuracy trade-offs will be reported at the end of the results section.

1. *Viewing eccentricity* Does the viewing eccentricity have an impact on response times?
  **Hypothesis**
  Yes, response times will be slower at higher eccentricities.
2. *Crowding* Does crowding impair response times?
  **Hypothesis**
  Yes, crowding should lead to impaired response times at smaller eccentricities than in condition 1 (no crowding).
3. *Expertise*: Are there response time differences between high- and low-skilled players in any of the four conditions?
  **Hypothesis**
  Yes, response time differences between groups are expected in conditions 3 and 4 but not in condition 1 or condition 2 as the latter two are very simplified tasks and do not require basketball-specific expertise.
4. *Viewing eccentricity × expertise*: Are viewing eccentricity effects on response times different between the two groups?
  **Hypothesis**
  Yes, high-skilled players but not low-skilled players will be able to compensate for eccentricity effects in condition 4 due to their better positioning and orientation skills.

## Results

### Condition 1

**Figure 1:**
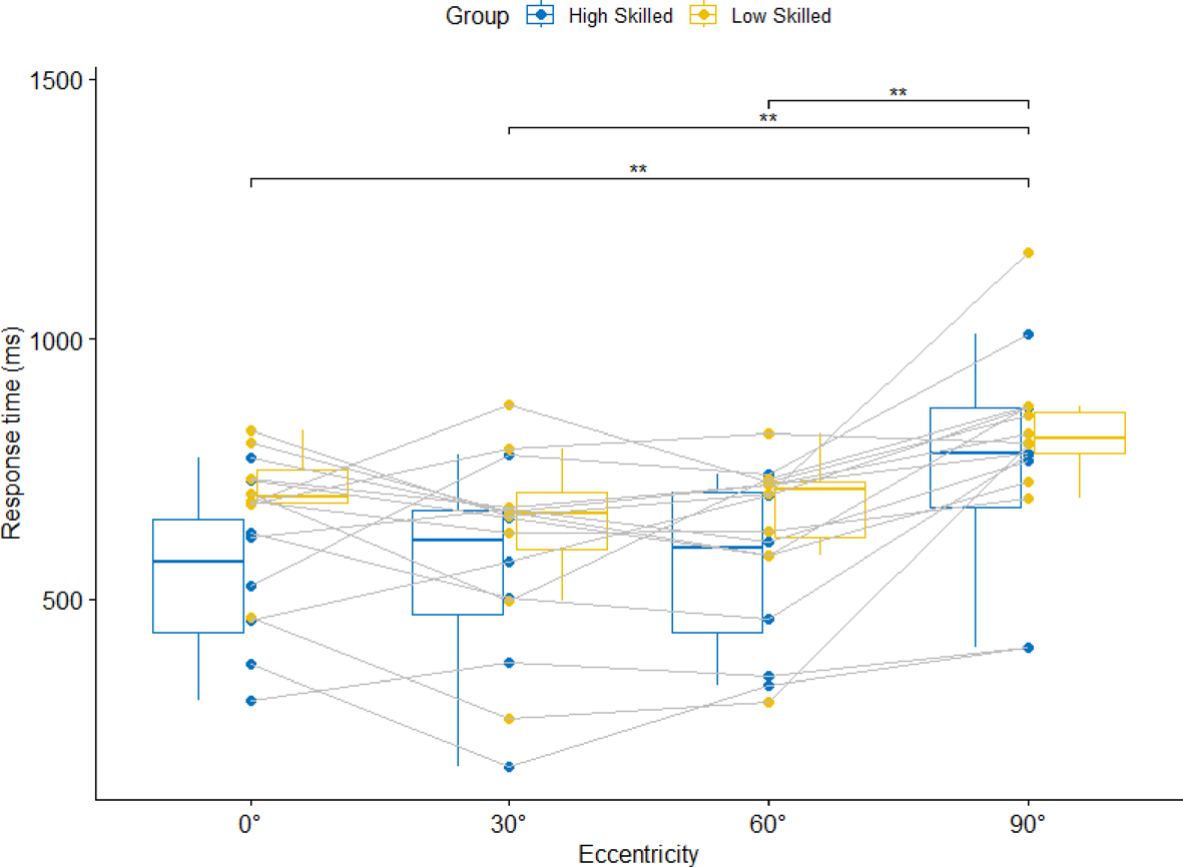
Median response times in condition 1 with four attackers and no defenders for the four different viewing eccentricities. Significant post-hoc differences between eccentricities are indicated by the horizontal brackets.

A significant main effect of Eccentricity was found for response time, *F*(2.5, 35.05) = 12.52, *p* < 0.001, η_p_^2^ = 0.47. Post-hoc follow-up tests indicated that response time was significantly higher at 90° eccentricity compared to 60°, 30° and 0° (see Fig. 1). There was neither a group effect, *F*(1, 14) = 2.13, *p* = 0.33, η_p_^2^ = 0.13, nor an interaction between group and eccentricity, *F*(2.5, 35.05) = 0.31, *p* = 0.79, η_p_^2^ = 0.02.

For response accuracies, there was neither an eccentricity effect, *F*(2.39, 33.44) = 1.96, *p* = 0.30, η_p_^2^ = 0.12, nor a group effect, *F*(1, 14) = 5.70, *p* = 0.10, η_p_^2^ = 0.29, nor an interaction between group and eccentricity, *F*(2.39, 33.44) = 1.22, *p* = 0.31, η_p_^2^ = 0.08.

### Condition 2

**Figure 2:**
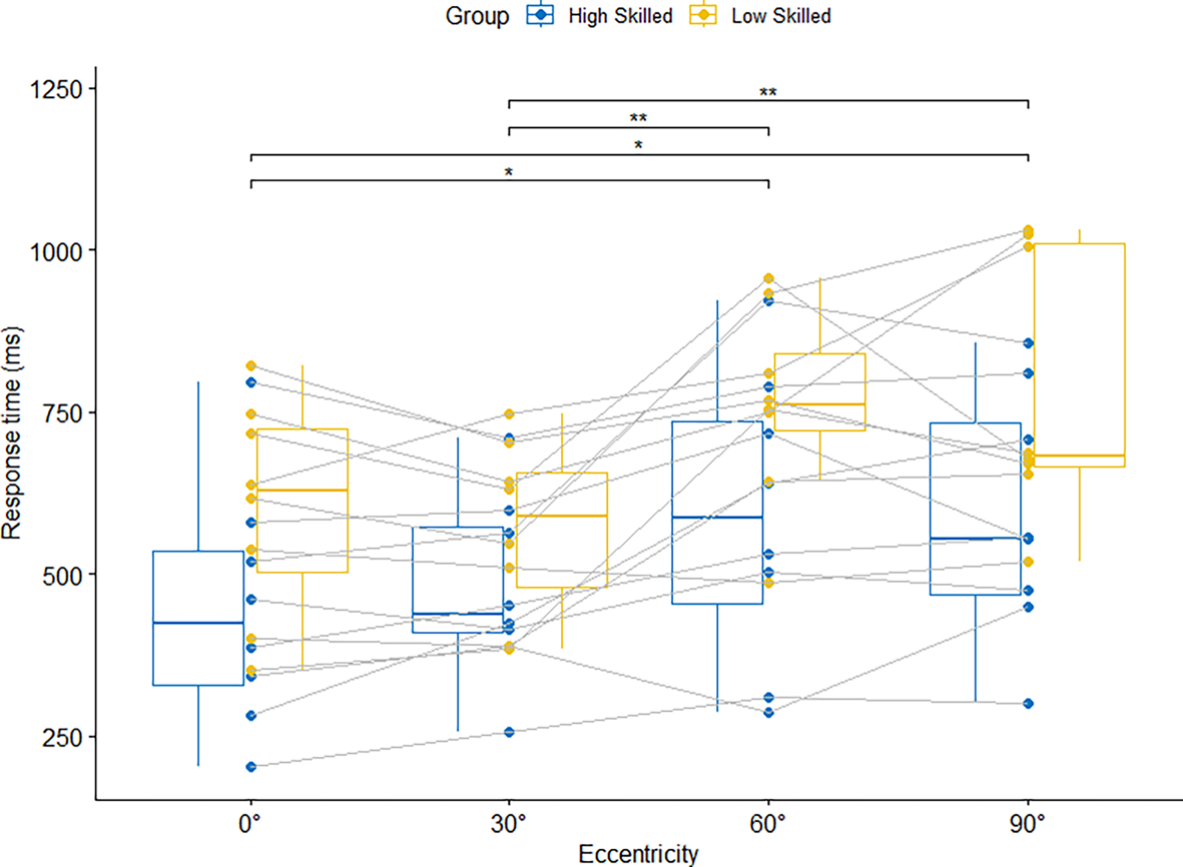
Median response times in condition 2 with four attackers and four defenders for the four different viewing eccentricities. Significant post-hoc differences between eccentricities are indicated by the horizontal brackets.

A significant main effect of Eccentricity was found for response time, *F*(1.94, 27.12) = 11.57, *p* < 0.001, η_p_^2^ = 0.45. Post-hoc follow-up tests indicated that response time was significantly higher at 90° and 60° eccentricity compared to 30° and 0° (see Fig. 2). There was neither a group effect, *F*(1, 14) = 4.20, *p* = 0.12, η_p_^2^ = 0.23, nor an interaction between group and eccentricity, *F*(1.94, 27.12) = 0.68, *p* = 0.51, η_p_^2^ = 0.05.

For response accuracies, there was neither an eccentricity effect, *F*(2.32, 33.53) = 1.04, *p* = 0.75, η_p_^2^ = 0.07, nor a group effect, *F*(1, 14) = 1.57, *p* = 0.69, η_p_^2^ = 0.10, nor an interaction between group and eccentricity, *F*(2.32, 33.53) = 0.82, *p* = 0.74, η_p_^2^ = 0.06.

### Condition 3

**Figure 3:**
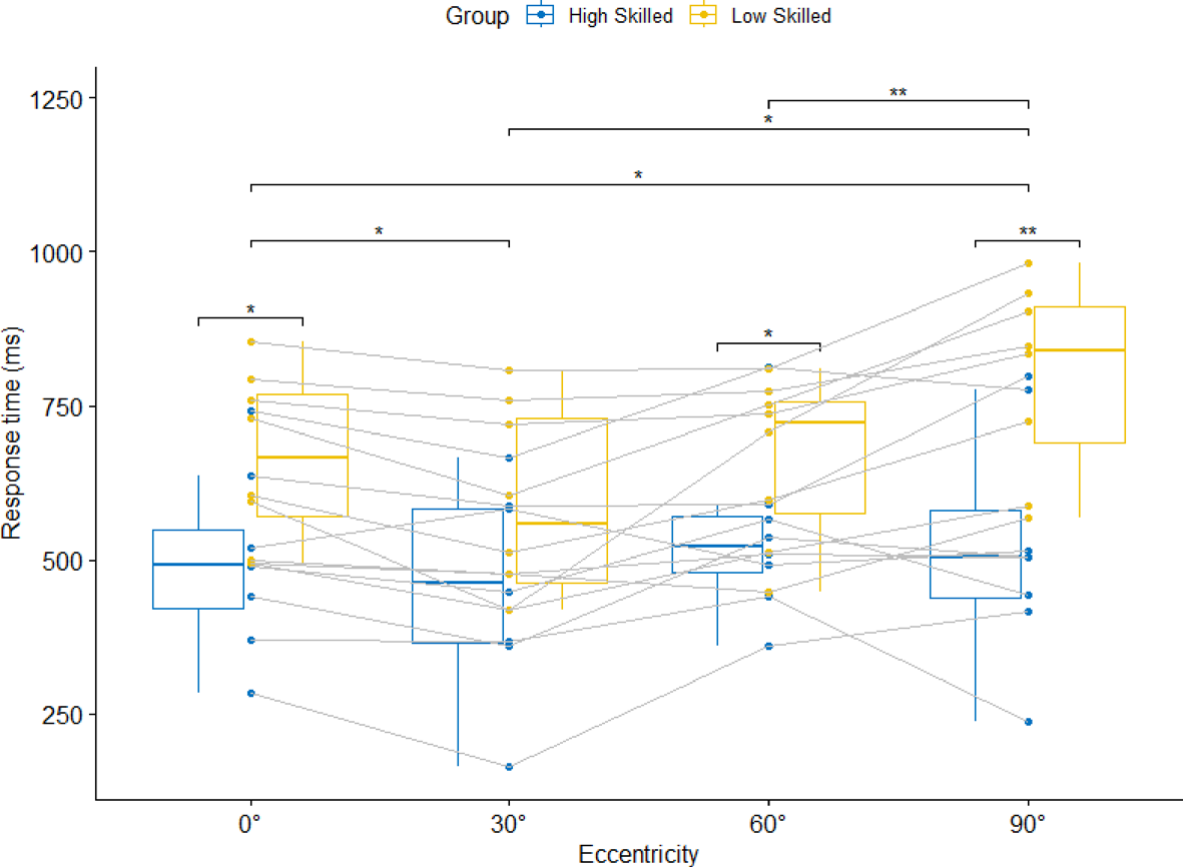
Median response times in condition 3 with four attackers and four defenders and the ball carrier eventually passing to a teammate. Data is presented for the four different viewing eccentricities.

There was a significant main effect of Eccentricity for response time, *F*(1.58, 22.08) = 11.95, *p* = 0.002, η_p_^2^ = 0.46. Response times were significantly higher at 90° compared to 30° and 0°. There was also a group, *F*(1, 14) = 6.31, *p* = 0.05, η_p_^2^ = 0.31, and an interaction effect between Group and Eccentricity, *F(*1.58, 22.08) = 4.11, *p* = 0.05, η_p_^2^ = 0.23. The group effect indicates faster response times of the high-skilled compared to the low skilled group. The interaction effect indicates that the group differences are only statistically significant at 0°, 60° and 90°, with the largest differences at 90° (see Fig. 3).

For response accuracies, there was an eccentricity effect, *F*(2.26, 31.68) = 10.49, *p* < 0.001, η_p_^2^ = 0.43. Response accuracy was lower at 90° eccentricity compared to 0° (*p* < 0.01), 30° (*p* < 0.01) and 60° (*p* = 0.02). There was neither a group effect, *F*(1, 14) = 0.58, *p* = 0.92, η_p_^2^ = 0.04, nor an interaction between group and eccentricity, *F*(2.26, 31.68) = 0.15, *p* = 0.92, η_p_^2^ = 0.01.

### Condition 4

**Figure 4:**
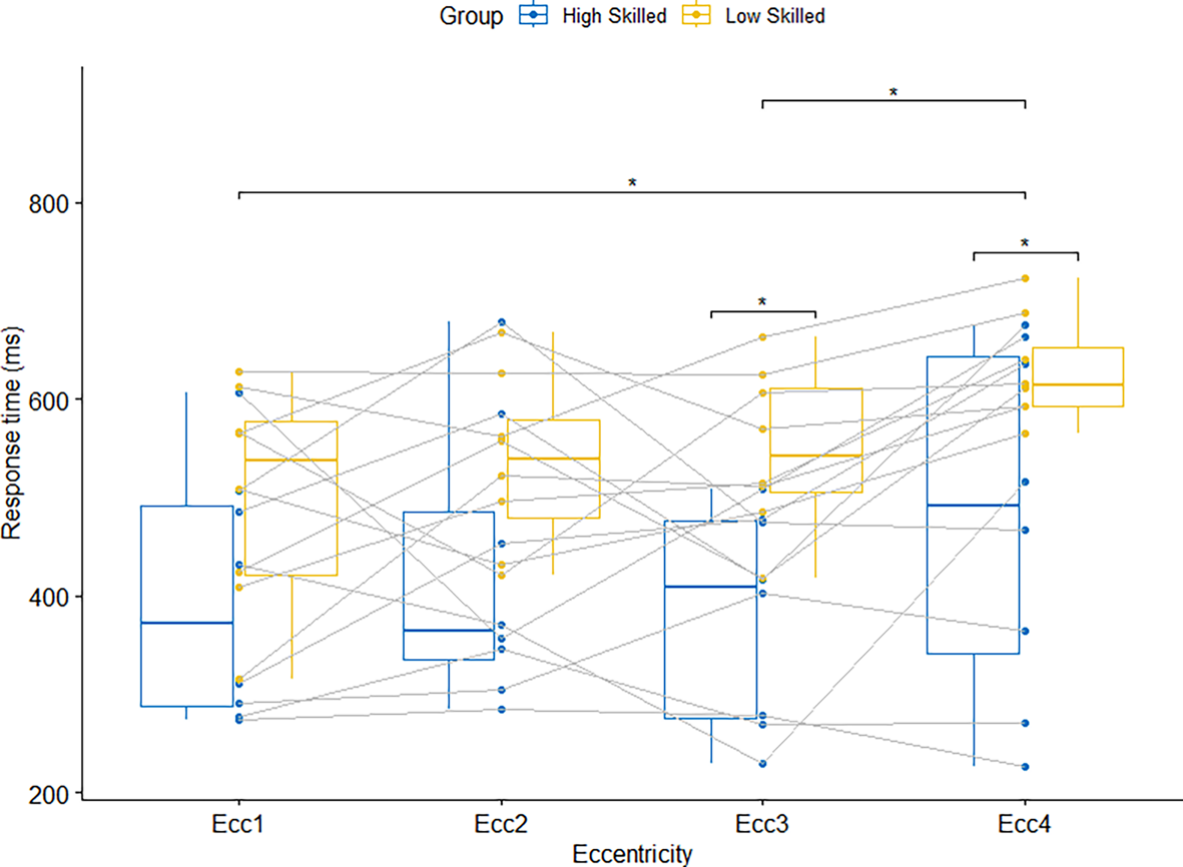
Median response times in condition 4 with simulated game situations for the two groups and the four eccentricities. Because players changed positions frequently, we report eccentricities relative to the ball carrier (Ecc1 means that the ball carrier cut to the basket; Ecc4 means that the player farthest from the ball carrier cuts to the basket).

There was a significant effect for Group, *F*(1, 14) = 8.43, *p* = 0.02, η_p_^2^ = 0.38, indicating that higher-skilled players responded faster than lower-skilled ones across all eccentricities (see Fig. 4). There was also a main effect of Eccentricity, *F*(2.77, 38.77) = 4.95, *p* = 0.02, η_p_^2^ = 0.26, which indicated faster response times at eccentricity 4 compared to 1 and 3 while the difference to eccentricity 2 failed to reach a significant difference (*p* = 0.24). There was no interaction between Eccentricity and Group, *F*(2.77, 38.77) = 0.52, *p* = 0.66, η_p_^2^ = 0.04.

For response accuracies, there was an eccentricity effect, *F*(2.54, 35.49) = 8.13, *p* < 0.01, η_p_^2^ = 0.37. Response accuracy was lower at Ecc4 compared to Ecc1 (*p* < 0.01) and Ecc2 (*p* < 0.01). There was neither a group effect, *F*(1, 14) = 0.76, *p* = 0.79, η_p_^2^ = 0.05, nor an interaction between group and eccentricity, *F*(2.54, 35.49) = 0.63, *p* = 0.79, η_p_^2^ = 0.04.

### Speed-accuracy trade-off in all conditions

Table 1 shows the response time and Table 2 the response accuracy results for all conditions, eccentricities and both groups.

**Table 1:** Means and standard deviations for response time (in ms) for all 4 conditions, 4 eccentricities and both groups.

Condition	Eccentricity	Group			
		High skilled		Low skilled	
		*M*	*SD*	*M*	*SD*
1	0°	551.97	165.34	697.24	108.25
1	30°	550.71	193.82	632.60	183.10
1	60°	563.76	162.13	651.80	156.71
1	90°	736.18	217.88	840.48	143.92
2	0°	445.92	187.55	603.79	165.48
2	30°	475.54	141.72	568.98	135.79
2	60°	587.18	223.55	761.87	150.98
2	90°	588.44	190.37	783.23	203.25
**3**	**0°**	**496.90**	**144.37**	**665.90**	**136.96**
3	30°	456.28	160.56	589.66	156.15
**3**	**60°**	**537.67**	**132.97**	**666.66**	**132.12**
**3**	**90°**	**524.85**	**185.14**	**797.59**	**155.22**
4	Ecc1	398.22	127.32	503.79	110.85
4	Ecc2	422.41	140.46	535.66	86.83
**4**	**Ecc3**	**382.33**	**108.30**	**549.04**	**81.31**
**4**	**Ecc4**	**477.49**	**176.88**	**628.46**	**52.82**

*M* and *SD* represent mean and standard deviation, respectively. Significant differences between groups are highlighted in bold (see results for statistics).

**Table 2:** Means and standard deviations for response accuracy (in %) for all 4 conditions, 4 eccentricities and both groups.

Condition	Eccentricity	Group			
		High skilled		Low skilled	
		*M*	*SD*	*M*	*SD*
1	0°	76.04	16.93	89.58	10.68
1	30°	91.67	8.91	91.67	10.91
1	60°	81.25	13.91	90.62	9.38
1	90°	72.92	24.70	89.58	9.71
2	0°	90.62	12.15	91.67	14.09
2	30°	81.25	18.77	91.67	13.36
2	60°	89.58	13.18	91.67	11.79
2	90°	78.12	22.69	90.62	12.15
3	0°	79.17	13.36	83.33	15.43
3	30°	80.21	20.38	86.46	9.90
3	60°	71.88	19.38	73.96	16.93
3	90°	54.17	25.20	62.50	21.36
4	Ecc1	88.42	10.01	79.78	20.73
4	Ecc2	82.74	11.08	83.52	10.66
4	Ecc3	76.97	7.41	74.17	10.60
4	Ecc4	71.43	6.23	65.94	18.01

*M* and *SD* represent mean and standard deviation, respectively. There was no significant difference between groups in any of the conditions or eccentricities.

**Figure 5:**
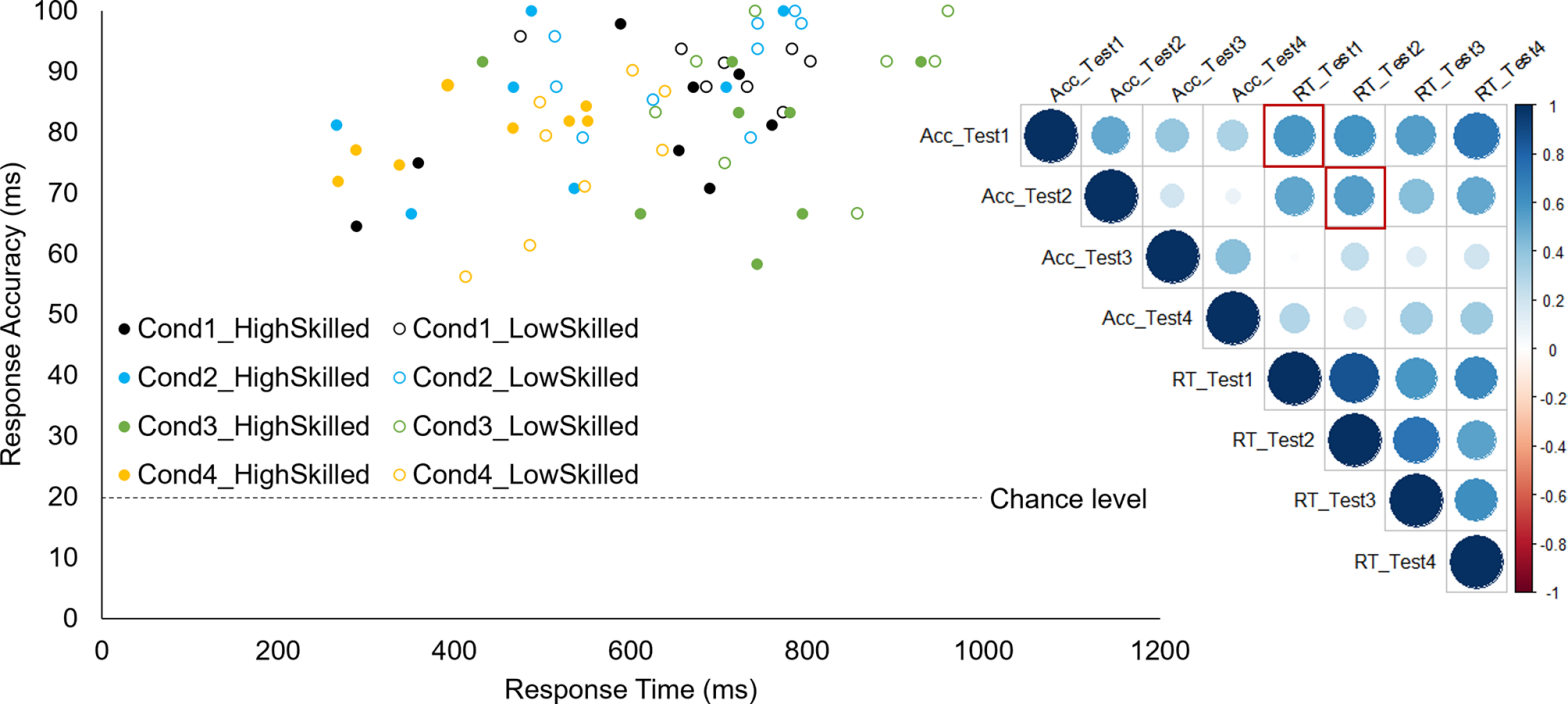
Relation between response accuracy and response time for the four test conditions (colours) and two groups (high skilled: filled circles, low skilled: empty circles). A Pearson correlation matrix is displayed on the right. The two significant positive correlations reported in the text are highlighted with a red square.

There was a speed-accuracy trade-off in condition 1 with a strong positive correlation (*r* = 0.58, *p* = 0.02) indicating that performance accuracy is positively correlated with response time. The same strong correlation (*r* = 0.56, *p* = 0.03) was found in condition 2. There was no correlation in condition 3 (*p* = 0.59) and 4 (*p* = 0.17; Fig. 5). Please note that the number of participants for such a correlation should optimally be higher and the results here should be treated as descriptive^36^.

## Discussion

This study is the first that tested the capabilities of using peripheral vision of sports athletes at large eccentricities in a controlled but representative experimental setting. The results show that high and low-skilled basketball players can detect the cut of players to the basket at up to 90° and without response time impairments at up to 60° eccentricity (condition 1). If the cutting player is crowded by a defender, response time performance is already impaired at 60° eccentricity (condition 2). When switching of attention from one player to another is required, high-skilled players can react faster than low-skilled players at 90° eccentricity (condition 3). When natural game-like decision making is demanded, high-skilled players respond faster at all eccentricities (condition 4).

Typically, peripheral vision performance is assessed with standardized test batteries like the peripheral vision test on the Vienna Test System, where LED lights need to be detected at different eccentricities^26,37^. In some studies that used the Vienna test system, skilled players were found to have faster reaction times than less-skilled players^38,39^ in another study there was no difference between skill groups^28^. So far, it was unclear if these findings also hold for sport-specific measurements^37^. In this study, we were able to create a basketball-specific peripheral vision test. Our results in condition 1 confirm our prediction that high- and low-skilled groups were able to process the cut of an attacker at eccentricities up to 90°, without any skill-group differences. Moreover, response times at 60° and 30° eccentricity were not different to those at 0° eccentricity, where players directly look at the player cutting to the basket. Only at 90° eccentricity, which is from the extreme corner of the eye, response times are slower compared to the other three eccentricities. Thus, it must be questioned if previous findings with general peripheral vision assessments can be translated to the sports context^40^.

When simulating a peripheral crowding by adding defence players in condition 2, results show—as hypothesized—that response times already increase at 60° in both groups. Thus, response times are impaired earlier with compared to without crowding (condition 1 vs. 2). This is the first study that showed that peripheral crowding impacts performance in a dynamic sport-specific task. From a research perspective, this underlines the need to control for peripheral crowding in future studies. From a practice perspective, these reaction time delays that arise through crowding should be considered in training. One way could be to adjust the help defence to a closer position under the basket to gain more time until the attacker needs to be stopped. That there is again no group effect underlines, that it is not the sport-specific expertise that explains the crowding effects but rather the perceptual capabilities of all players.

The first expertise effects are visible in condition 3, where a choice reaction was demanded. Results show that high-skilled players responded faster than low-skilled players in three out of four eccentricities, just as was hypothesized. One reason is that the demands in this condition are very close to the game. Compared to conditions 1 and 2, the actions of multiple players must be monitored at the same time because the player in possession of the ball could eventually play a pass to another player. The largest difference between both groups is evident at 90° eccentricity, which underlines that peripheral vision must play an important role in basketball defence. The result, that there was no response accuracy difference between groups, can be explained by the faster response times of the high-skilled group. Due to their earlier decisions, they accumulated less visual information than the low-skilled group. That response accuracies increase if more information can be processed has been shown in temporal-occlusion studies^41,42^.

The most representative task design is achieved in condition 4, where multiple passes are played, typical tactical offence and defence plays are included, and the participants are allowed to use their preferred positioning and gaze behaviour. In addition, the realistic 360° videos include representative motion cues and game patterns that are more effectively used by skilled compared to less-skilled athletes as reported for various perceptual-cognitive skills^3,35^. All of that is likely to lead to advantages for the high-skilled group, which indeed was visible in the data. High-skilled players responded faster than their low-skilled counterparts at all eccentricities. Besides this, response times for players cutting from Ecc4 are slower than response times at Ecc1, where the ball carrier cuts to the basket. This eccentricity effect might be related to the use of foveal (Ecc1) and peripheral (Ecc4) vision because previous research has indicated that basketball players look mostly to the player in ball possession^17,18^. Thus, it might be the case that high-skilled players are better able to use their foveal and peripheral vision.

These findings can have an impact on vision training in basketball and other game sports. Recent reviews on peripheral vision training tools either indicated that the degree of peripheral vision usage was either not controlled^26^ or there is not enough empirical evidence that shows transfer effects from sport-unspecific paradigms, like the Multiple Object Tracking paradigm, where multiple moving objects need to be monitored, to the game^43^. The representative task design used in this study might be better suited for training peripheral vision skills.

The findings can also have theoretical implications in the context of peripheral vision usage in (team) sports. First of all, the expertise findings show that an optimal orientation of attention can lead to response time advantages in visually complex decision-making scenarios^2^. Because of the eccentricity findings, a gaze anchor that reduces gaze angles to relevant areas of interest, might indeed be optimal in tasks that require fast responses^2,25^. Besides this, the results emphasize the need for representative task designs in studies that want to capture or train the perception–action coupling in team sports and increase the likelihood of skill transfer to the field^40,43^. It should be noted that instructing participants where to look, as was done in condition 1 to 3 to control visual eccentricities, might hinder experts to use their natural perceptual strategy. That means, gaze feedback may not always be appropriate to improve performance^44^. Athletes have different visual skills and perceptual strategies and breaking those might impair performance. Thus, theoretical concepts on peripheral vision should distinguish between vision (knowing where to look) and attention (knowing what to attend to).

Some limitations must be acknowledged. First, eye-tracking was not used to control the gaze location. That means, participants could have held their head straight but looked at other players than instructed in conditions 1 to 3. Previous research has shown that head and eye movements are typically coordinated and occur together^45^. Thus, it is likely that, especially at the large eccentricities, participants would have reoriented their head to detect the onset of a cut, which would have been detected with our head-motion manipulation check. To achieve this extra gaze control, future research might profit from recent developments in eye-tracking hardware. The Pupil Labs Neon glasses, as an example, might be suitable for future research as there is a version (called: “Is this thing on?”; https://pupil-labs.com/products/neon/) that does not block peripheral vision, which most other devices do. A second limitation of this study is the rather small sample size. While the sample size was calculated a priori, it was only possible to refer to an effect size from a highly controlled experimental MOT study. While the results of this study also show high eccentricity effect sizes, there is still the need for replication. Third, it was decided to not counterbalance the test conditions to increase task complexity from condition 1 to condition 4 for all players in the same way. A comparison of performance between conditions is likely influenced by learning effects in the current design. Counterbalancing the conditions would have solved this problem. Moreover, response times in condition 4 cannot be compared to response times in conditions 1 to 3, because the intention of a player, to cut to the basket, can be identified by “reading the play”, which is also the case in the real games. However, this was not possible in the other conditions of this experiment.

In conclusion, the results show that viewing eccentricity matters when quick reactions are required in basketball defence. It can be advantageous to locate relevant players closer to the fovea to detect the cutting action earlier and respond with a defensive movement in time. A second finding is that expertise effects in response time can only be found if the task design is more representative (e.g., in terms of the complexity of the game, visual cues and playing patterns) and the experimental scenario mirror the demands of the actual game, namely if they require to distribute attention to multiple locations. Third, the results of conditions 1 and 2 emphasize that skilled players might not have better peripheral visual skills than low-skilled players when simple reactions are demanded. Finally, visual crowding not only affects response times in basic science but also in a basketball decision-making task. These findings can be used to design appropriate peripheral vision exercises, where player positions are systematically varied at different eccentricities. Such training is likely to improve response times in a real game because our high-skilled players—who presumably were more familiar with players cutting to the basket from different eccentricities—responded faster than the low-skilled players.

## Method

### Participants

An a priori G*Power^46^ calculation for the required sample size given an α of 0.05, a power of 0.95 and a large effect size of f = 0.4 (based on previous crowding effects on tracking accuracy results in^47^), with two groups (high- and low-skilled), four measurements (eccentricity), and a correlation among repeated measures of *r* = 0.5, revealed a required sample size of 16 participants. Thus, 16 male basketball players (*M* age = 23.9 ± 4.2 years) were included in this study. Eight players were included in the high-skilled group (*M* age = 26.3 ± 4.2 years) and had 13.8 (± 5.3) years of playing experience in one of the four highest leagues in Switzerland. The low-skilled group (*M* age = 21.5 ± 2.2 years) were recruited from the university sport of the local university where they played basketball for on average of 3.25 (± 3.3) years. All the players had experience in playing the centre position. All participants reported normal or corrected-to-normal vision. Ethical approval for the study was obtained from the ethical commission of the Faculty of Human Sciences of the University of Bern. The study was performed in accordance with faculty’s guidelines and regulations, which agree with the Declaration of Helsinki (2008).

### Stimuli and experimental conditions

Video stimuli were recorded in a local sports hall. A GoPro Omni (Woodman Labs, Inc., San Mateo, USA) with six synchronized GoPro Hero 4 black (60 Hz) producing a spherical video array was used. The Omni was mounted onto a tripod and placed directly under the basket at a height of 1.60 m (the approximate height of a 1.85 m player when in a defence position with bended knees). The cameras were synchronized by an auditory signal (one player clapped his hands). Eight experienced basketball players, who provided informed consent to use video images in this publication, staged the situations for four different experimental conditions. The video footage was rendered into a spherical 360° movie (Autopano Video Pro, Kolor SAS, France). Unwanted noise was extracted and the visible tripod the tripod removed with a fitting environment (Adobe Premiere Pro CC, Adobe Systems Software, Ireland). The situations were then cut into single experimental trials. The first frame of every trial was frozen for 3 s to give participants the time to view the players’ positions before they started to move.

**Figure 6:**
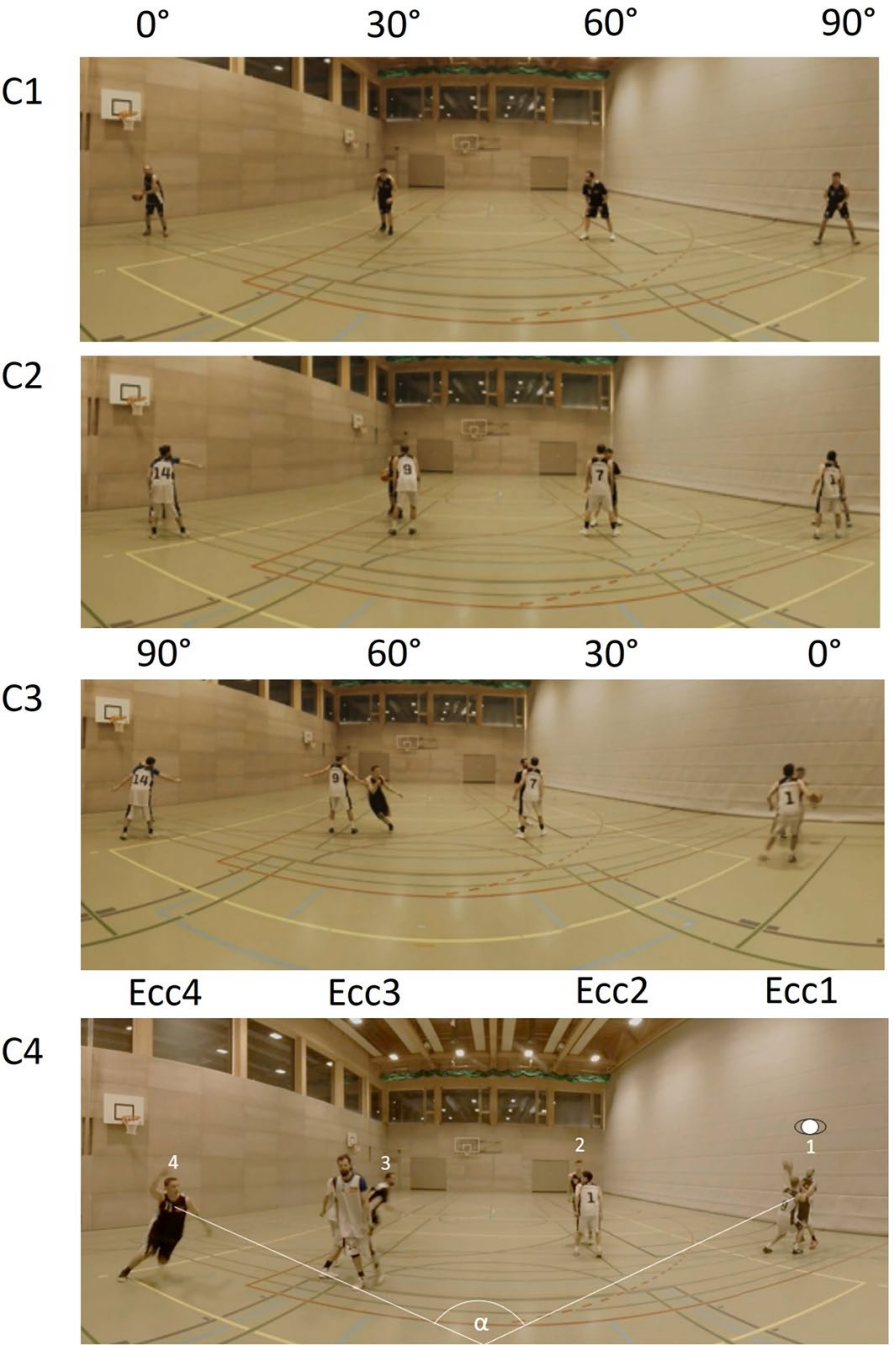
The four experimental conditions that required simple reactions without crowding (**C1**), simple reactions with crowding (**C2**), choice-reactions with crowding (**C3**) and reactions in game-like scenarios (**C4**). While visual eccentricities were controlled in condition 1 to 3, this was not possible in condition 4, because often changed their positions on the field, set blocks for other players or played sets like pick-and-roll, just as in the game. Thus, Ecc1 to Ecc4 refers to the player's eccentricity relative to the ball. The angle α represents the viewing angle between the ball carrier and the cutting player, which is still around 90° in the example image in condition 4.

Condition 1 included only the four attackers. The scenario aimed to stage situations where either the ball carrier or one of the other players without the ball cut to the basket. The three players were positioned at relative viewing angles from the ball carrier without defenders. Since peripheral vision is sensitive to crowding (see introduction), this condition should be used to test reaction times in uncrowded conditions. When the participant would look at the ball carrier, the other players were positioned at 30°, 60°, or 90° eccentricity (Fig. 6, C1). In every trial of this condition, either the ball carrier or one of the other players cut to the basket. The players were instructed before each trial, on which of them should start the sprint to the basket after the experimenter clapped in his hands. If it was the ball carrier himself, he started dribbling to the basket. If it was another player, he started sprinting and received the ball from the ball carrier. The direct defender was later simulated with a stick in the laboratory so that a 4-outside-1-inside formation was created. The direct defender was later simulated with a stick in the laboratory so that a 4-outside-1-inside formation was created. In total, 48 scenes were recorded (12 for each eccentricity).

**Figure 7:**
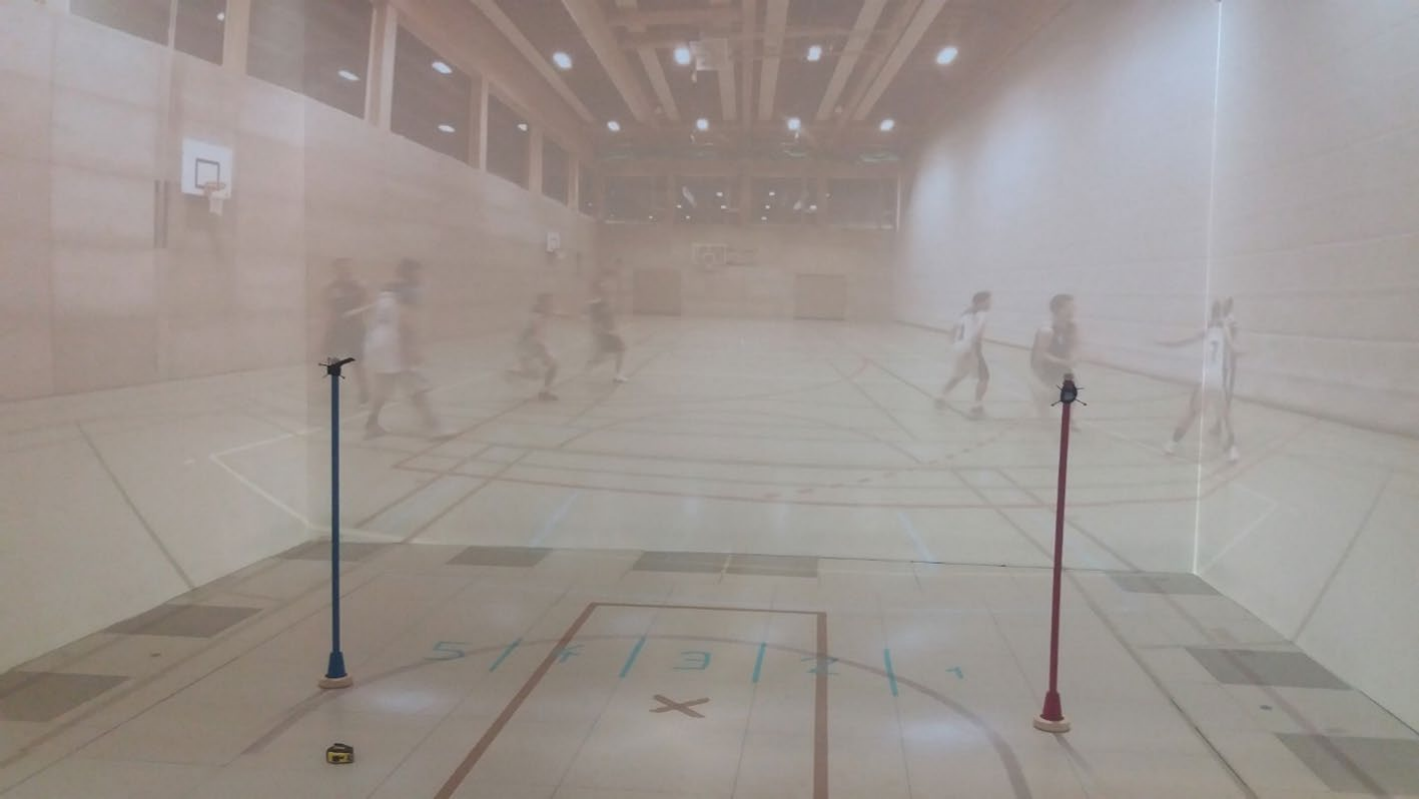
The laboratory with the CAVE and the 360° videos projected onto the walls and the floor. The poles indicate the position of the direct opponent of the participant being either on the left or right side of the basket depending on the ball position. The floor markings indicate the participants' starting position (“x”) and five potential locations for defending position (numbers 1–5). The participant had to react as fast as possible to a cutting player. Both poles were equipped with an Optitrack cluster to also analyse the participants’ position with the Motion Tracking System.

In condition two, defenders were included at the four eccentricities to have a natural simulation of a real game with four attackers and four defenders outside (Fig. 6, C2). The direct defender was later simulated with a stick in the laboratory (Fig. 7) so that a 4-outside-1-inside formation was created in the lab. The defenders were instructed to let the attacker pass and only defend with 50% intensity. In total, 48 scenes were recorded (12 for each eccentricity).

In condition three, the ball carrier always cut to the basket and either attempted to score a basket on his own or passed the ball to another player who cut to the basket (Fig. 6, C3). This condition was implemented to test the choice reaction times when the focus of attention must be switched from one player (the ball carrier) to another player (at different eccentricities. In 25% of the scenes, the defender let the player with the ball pass and in 75% the ball carrier played a pass to one of the other players. In total, 48 scenes were recorded (12 for each eccentricity).

In condition 4, players were instructed to simulate a natural gameplay, change positions, and play different attacking sequences with a variety of tactical elements (e.g., pick and roll, give and go, backdoor cuts; Fig. 6, C4). Players had to play four to ten passes in each scene before a player cut to the basket to vary the length of the situations before one offensive player would cut to the basket. The player cutting to the basket, and his relative position to the ball carrier varied across trials to manipulate visual eccentricity. This led to four different cutting eccentricities (eccentricity 1: the player with the ball cuts to the basket; eccentricity 4: the player who is furthest from the player with the ball cuts to the basket, see Fig. 6, C4). Two research assistants marked the eccentricities. In total 21 scenes were recorded for each eccentricity (84 scenes in total). Two scenes, both for eccentricity 2, had to be excluded because one GoPro Camera did not record the scene correctly so that 82 trials were shown to the participant. The scenes from condition 4 were already used in a previous study^48^.

### Equipment and motion analyses

A life-sized Cave Automatic Virtual Environment (CAVE) environment was used to project the basketball situations on a 6.00 m × 3.75 m front wall, two 11.00 m × 3.75 m sidewalls, and the 6.00 m × 11.00 m floor. The CAVE consisted of 12 cluster workstations (each with a Nvidia-Quadro-A6000 graphics card) and 12 projectors (Barco F50, 2560 × 1600, 60 Hz). The cave was equipped with a 24-camera Optitrack system (Prime 17 W, Prime 13 W, 240 Hz, 1,3 MP) to track retroreflective markers or cluster markers on the participant’s body.

This system was prepared before the first participant of the day. To achieve this, all retroreflective material was removed from the CAVE before the cameras were masked to avoid any unwanted reflections. Next, the aiming procedure started by waving a stick with retroreflective markers with standardized marker distances through the entire CAVE volume to achieve maximum data collection precision. The calibration finished when all cameras received at least 3000 samples and the calibration was rated at least “excellent”. Last, we set the volume origin by putting the calibration stick in a standardized location in the middle of the room and manually corrected the origin of the calibration to be in the bottom left corner of the front wall.

Four Optitrack clusters were used to capture the location and head orientation of the participant. The clusters were attached to the right foot, the left foot, the hip and the head. The foot clusters were attached with a strap close to the ankle. The hip cluster was attached to a belt at the back of the participant and the head markers were on a headband. Each cluster contained four spherical reflectors. It was guaranteed that at least three were always detected by the Optitrack system to ensure accurate tracking. To simulate the position of the centre player, poles were positioned at a 1.25 m distance to the right and left of the starting position of the participant. To locate the position of the poles in Optitrack, marker clusters were attached at the top of each pole. To help the players report their final position, we prepared five floor markings. The distance between each of the markings was 50 cm (i.e., each response fields was a 50 cm × 50 cm square). The starting position was 50 cm behind the middle of response field number 3 (see Fig. 2).

Motion data of all markers was captured at 200 Hz and was analyzed using Matlab 2022a. After importing the raw data for all four body clusters, a low pass filter with a window size of 20 frames was used to interpolate the data. Next, the position data was integrated (divided by the frame rate) to obtain velocity data. This data was then integrated another time to receive acceleration data. A third-order Savitzky–Golay filter with a window size of 15 frames was then applied to the integrated data. The foot movement was detected when either foot exceeded a 5 m/s threshold in the forward direction (towards the frontal screen). A backward-in-time search to a velocity smaller than 0.25 m/s was used to detect the onset of the foot movement.

### Procedure

After signing informed consent, participants were equipped with the four Optitrack clusters (head, hip, right foot, left foot). Next, participants were positioned at a 3 m distance from the central wall and 3 m from the right and left wall (see Fig. 7). All clusters were labelled and calibrated in Motive (Optitrack Software) while the participant was standing in a T-pose. Next, the head orientation was calibrated. For this, the participant was asked to stand still, orient the body and head towards the center of the front wall and look at a fixation point which was projected at eye-level of the participant onto the front wall. This calibration was checked before every block of trials. A re-calibration was conducted in case the head orientation deviated by more than 20 cm (i.e., approx. 4° visual angle). from the calibration point.

Each condition started with three (condition 1 and 2), five (condition 3) or six (condition 4) practice trials. The number of practice trials was increased in conditions 3 and 4 because of the increased complexity of the stimuli. All participants completed the conditions in the same order (condition 1, condition 2, condition 3, condition 4). The reason for not counterbalancing the conditions was, to have the “perimeter” (vision) test first and assess how well participants could react to the easiest (visual) condition without crowding and subsequently increase the visual and motor demands and with that the task representativeness. The experiment lasted about 120 min per participant including short breaks between the conditions.

### Measures

Response accuracy was defined as the percentage of trials where the participant stepped onto the correct response field, which was defined by a basketball coach with playing experience in the first national league of Switzerland. In some cases, two fields were accepted as correct. The trial was correct if the participant’s final position (i.e., the mean position of both foot clusters) landed on the fields that were identified as correct by our expert.

Response time was defined as the time between the onset of a player's cut, defined as the first body movement towards the basket, until the onset of the participant's foot movement towards the front wall in conditions 1 to 3 (see chapter “Equipment and Motion Analyses”). In condition 4, we defined the onset of a player's cut as the time point when an offensive player beat his defender defined as the moment when he was closer to the basket than the defender. As a consequence, response times will presumably be faster in condition 4 compared to 1–3 because the cut motion can be detected before the player beats his defender.

Head orientation was used as a control variable to check that participants oriented their head towards the instructed player. This is necessary to achieve the manipulated viewing eccentricities. It was decided to not use eye-tracking—which would have allowed an even more accurate manipulation of the location of gaze rather than the head—because the frame of any eye-tracking glasses would have blocked the visibility of players in the periphery. Instead, it is known, that head movements are suited to control for eye-movements because head movements and saccades are linked^45,49,50^. Humans try to keep the eye orientation in a “neutral” position roughly centred in the orbit and even targets at 20° eccentricity lead to head re-orientations^49^. In this experiment, the correct head orientation was checked with the head cluster. The marker positions were used to calculate a vector from the central forehead to the front wall. The intersection between this vector and the front wall was calculated. Afterwards, the maximum deviation of this intersection from the start of the trial until the onset of a player’s movement in the video was calculated. We defined a maximum distance of 20 cm between the head intersection and the position of the instructed player for a successful manipulation check (approx. 4° visual angle). Trials where this distance was exceeded were excluded from the analyses (0.98% of the trials had to be excluded). Given that the minimum eccentricity in condition 1 to 3 was 30° from one player to another and head movements already occur at eccentricities greater than 20°, the likelihood of detecting a saccade by checking the head amplitude is very high. Therefore it was decided to use the head orientation as a manipulation check rather than eye-tracking devices. Additionally, the eye-tracker would have blocked peripheral vision (and the stimuli), which would have led to changes in eye- and head-movements^17^.

### Analyses

Decision accuracy and reaction times were analyzed for each experimental condition with a 4 (Eccentricity: 0°, 30°, 60°, 90°) × 2 (Group: high-skilled, low-skilled) repeated-measures ANOVA with Eccentricity as the within- and Group as the between-group factor. Because of the directed hypotheses that reaction times increase with eccentricity and high-skilled react faster than low-skilled players, one-tailed paired *t*-tests were calculated as planned contrasts in case the ANOVAs revealed significant effects. Effect sizes were computed as partial eta squared (η_p_^2^) or Cohen’s *d*. Additionally, to identify a speed-accuracy trade-off in any of the four conditions, Pearson correlations between response accuracy and response time are reported in a correlation matrix^51^. All statistical analyses were performed using *R*. The homogeneity of variance of the between-subject factor was checked using Levene’s test and the sphericity of the within-subject factor with the Shapiro–Wilk test. The “afex”-package^52^ was used for repeated-measures ANOVAs and the “emmeans”-package^53^ for post hoc contrasts. The *p* values of the ANOVAs were adjusted with the Holm method and in case the sphericity assumption was violated, Greenhouse–Geisser corrections were applied. Post hoc comparisons were Bonferroni adjusted. Plots were generated using the “ggpubr”-package^54^. The boxplots show the median of all participants for each condition as well as the 25th and 75th quartile. Error bars represent 1.5 times the upper or lower interquartile range. The horizontal brackets indicate significant differences between conditions (levels of significance: *p < 0.05; **p < 0.01). Individual data points will be connected with grey lines to visualize individual performance changes over the conditions.

## Data Availability

The data that support the findings of this study are openly available on Open Science Framework (OSF) at this link [10.17605/OSF.IO/4EPT8].
